# Carnosic acid mitigates doxorubicin-induced cardiac toxicity: Evidence from animal and cell model investigations

**DOI:** 10.22038/IJBMS.2023.71508.15544

**Published:** 2024

**Authors:** Mahboobeh Ghasemzadeh Rahbardar, Farhad Eisvand, Maryam Rameshrad, Bibi Marjan Razavi, Abbas Tabatabaee Yazdi, Hossein Hosseinzadeh

**Affiliations:** 1 Pharmaceutical Research Center, Pharmaceutical Technology Institute, Mashhad University of Medical Sciences, Mashhad, Iran; 2 Department of Pharmacodynamics and Toxicology, School of Pharmacy, Mashhad University of Medical Sciences, Mashhad, Iran; 3 Targeted Drug Delivery Research Center, Pharmaceutical Technology Institute, Mashhad University of Medical Sciences, Mashhad, Iran; 4 Ghaem Hospital, Department of Pathology, Mashhad University of Medical Sciences, Mashhad, Iran

**Keywords:** Anti-Inflammatory agents, Anti-oxidants, Cardiotoxicity, Electrocardiography, Salvin, Vitamin E

## Abstract

**Objective(s):**

Utilization of doxorubicin (DOX) as a chemotherapy medication is limited due to its cardiotoxic effects. Carnosic acid exerts antioxidant, anti-inflammatory, besides cytoprotective effects. The objective of this study was to investigate the ability of carnosic acid to protect rat hearts and the MCF7 cell line against cardiotoxicity induced by DOX.

**Materials and Methods:**

The study involved the classification of male Wistar rats into seven groups: 1) Control 2) DOX (2 mg/kg, every 48h, IP, 12d), 3-5) Carnosic acid (10, 20, 40 mg/kg/day, IP, 16d)+ DOX, 6) Vitamin E (200 mg/kg, every 48h, IP, 16d)+ DOX 7) Carnosic acid (40 mg/kg/day, IP, 16d). Finally, cardiac histopathological alterations, ECG factors, carotid blood pressure, left ventricular function, heart-to-body weight ratio, oxidative (MDA, GSH), inflammatory (IL-1β, TNF-α), plus apoptosis (caspase 3, 8, 9, Bcl-2, Bax) markers were evaluated. DOX toxicity and carnosic acid ameliorative effect were evaluated on MCF7 cells using the MTT assay.

**Results:**

DOX augmented the QRS duration, QA, RRI, STI, and heart-to-body weight ratio, and reduced HR, LVDP, Min dP/dt, Max dP/dt, blood pressure, boosted MDA, TNF-α, IL1-β, caspase 3,8,9, Bax/Bcl-2 ratio, decreased GSH content, caused fibrosis, necrosis, and cytoplasmic vacuolization in cardiac tissue but carnosic acid administration reduced the toxic effects of DOX. The cytotoxic effects of DOX were not affected by carnosic acid at concentrations of 5 and 10 μM.

**Conclusion:**

Carnosic acid as an anti-inflammatory and antioxidant substance is effective in reducing DOX-induced damage by enhancing antioxidant defense and modifying inflammatory signal pathway activity and can be used as an adjunct in treating DOX cardiotoxicity.

## Introduction

Doxorubicin (DOX), also recognized as adriamycin, is a principal and extensively-used chemotherapeutic agent separated from *Streptomyces peucetius* var. caesius and has been revealed to be greatly operative in the treatment of various human cancers (1), but its usage is restricted due to its adverse effects, particularly cardiotoxic effects, which recurrently result in heart failure and irretrievable degenerative cardiomyopathy (2). These unfavorable effects often arise from DOX cumulative doses (3). Multiple mechanisms of action have been announced for DOX, such as elevated oxidative stress and fundamental interaction with DNA (4). The literature has also suggested that apoptosis and inflammation are the two primary mechanisms involved in DOX-induced cardiotoxicity (5). Due to the heart’s less developed anti-oxidant defense mechanisms and increased sensitivity to free radicals, cellular damage brought on by DOX-induced oxidative stress may be greatly intensified (6). Likewise, free radicals are also responsible for the impairment of the myocardium after receiving DOX, and thus anti-oxidants might be beneficial to protect the heart after DOX exposure (7). At present, a few treatments are available for cardiomyopathy stimulated by DOX. Plant-derived or synthetic substances that can minimize or eliminate DOX-induced cardiac damage would be tremendously valuable for cancer treatment. Such medicines could help protect the heart from toxicity while still allowing greater DOX doses to be utilized effectively against malignancies. This may aid in improving outcomes by increasing the ratio of DOX’s anti-cancer properties to its unpleasant cardiac side effects.

Carnosic acid is a type of chemical molecule known as a benzenediol diterpene found in certain plants, such as common salvia (*Salvia officinalis*) and rosemary (*Rosmarinus officinalis*) in the Lamiaceae family (8, 9). This lipophilic molecule is well-known for its powerful anti-oxidant property, which has resulted in its extensive application in a variety of industries, including nutrition and health, food and beverage, and personal care (10). Previous studies have shown that carnosic acid can prevent the oxidation of low-density lipoprotein cholesterol in endothelial cells that line the inner walls of human aortic blood arteries (11). Carnosic acid increases the levels of endogenous enzymes that neutralize free radicals, thereby exerting its anti-oxidant properties. This is accomplished by stimulating the transcriptional process regulated by the nuclear factor erythroid 2-related factor 2 (Nrf2), which controls the expression of anti-oxidant genes and enzymes (12). Research findings consistently demonstrate that carnosic acid possesses anti-inflammatory properties, as evidenced by studies conducted in both laboratory (*in vitro*) and live organism (*in vivo*) settings. Carnosic acid was shown in mouse research to minimize inflammation in the ears caused by phorbol 12myristate 13acetate. The mechanism of action involves a decrease in the gene expression of pro-inflammatory cytokines, specifically interleukin 1 beta (IL-1β) and tumor necrosis factor-alpha (TNF-α). Furthermore, carnosic acid was discovered to reduce the excess synthesis of the signaling molecule nitric oxide in RAW 264.7 immune system macrophages (13). Another study found that carnosic acid could decrease inflammatory responses in skin tissue by lowering the amounts of pro-inflammatory cytokines, namely TNF-α and IL-6 (14). Additionally, carnosic acid has been illustrated to have a protective impact contrary to several tissue injuries through inhibiting inflammation and apoptosis (15-17). Liu and Dong also stated that carnosic acid inhibited the apoptosis process in H9c2 cardiomyocytes that occurred with hypoxia/reoxygenation by up-regulating B-cell lymphoma-2 (Bcl-2) level and attenuating Bcl-2 associated X (Bax) as well as caspase3 amounts (18). In a mouse model of myocardial stress, this compound manifested cardioprotective effects by suppressing apoptosis and oxidative stress (19).

The objective of this study was to examine the potential protective effects of carnosic acid against doxorubicin-induced cardiac toxicity by evaluating histological changes in heart tissue, various hemodynamic parameters (RR interval (RRI), Q amplitude (QA), heart rate (HR), QT, ST interval (STI), QRS voltage and duration, diastolic pressure, systolic pressure, derivative of pressure over time (dP/dt), mean arterial pressure (MAP), left ventricular developed pressure (LVDP), dP/dt Minimum (Min dP/dt), dP/dt Maximum (Max dP/dt)) by a Millar pressure catheter and electrocardiography, heart weight/body weight ratio, body weight, heart weight, oxidative stress factors (malondialdehyde (MDA), glutathione (GSH)), inflammatory markers amounts (TNF-α and IL-1β), and apoptosis (caspase 3, 8, 9, Bax, as well a Bcl-2) in the cardiac tissue. Our team also conducted *in vitro* tests using MCF7 breast cancer cells to evaluate how carnosic acid may impact DOX’s anticancer activity, as measured by cell viability assays.

## Materials and Methods

***Animals***

All animal research was performed following ethical standards and guidelines established by Mashhad University of Medical Sciences. The study was carried out in compliance with internationally recognized principles for the ethical treatment and appropriate use of laboratory animals.

The study used male Wistar rats weighing between 265 and 330 grams. The rats were housed in an animal house facility under controlled conditions, including a 12-hour light/dark cycle and a consistent temperature of 22±2 ^°^C. Throughout the study period, the rats were provided with *ad libitum* access to both food and water. Animal discomfort was minimized, and the number of rats utilized in various tests was limited.

***Materials***

The used materials were as follows: DOX (Ebewe-Austria); carnosic acid (Sigma Aldrich, St. Louis, MO, USA.); heparin and Vitamin E (Tehran Darou Pharmaceutical Co.); Xylazine and ketamine (Alfasan Pharmaceutical Co., Woerden, Netherlands). 

***Drug administration***

The trials were conducted during the day (from 8:00 to 13:00) in a peaceful atmosphere. The study involved the random assignment of 42 rats into seven distinct groups, with six rats allocated to each group. Moreover, the medicines used in the trial were freshly produced. Carnosic acid was administered intraperitoneally (IP) at different doses (10, 20, and 40 mg/kg), based on previous studies (20). In an acute toxicity experiment conducted in mice, the investigation determined that the oral administration of carnosic acid resulted in a lethal dose of 7100 mg/kg in the experimental subjects (21). The rats were administered therapeutic doses of carnosic acid 30 min prior to the injection of DOX. It should be noted that carnosic acid was dissolved in a mixture of normal saline and Tween 80%, but DOX was dissolved in normal saline.

***Study protocol***

Group 1: control Group: Animals in this group were administered 0.5 ml of vehicle (normal saline) and 15 µl of Tween 80% via intraperitoneal (IP) injection.

Group 2 received an IP injection of DOX at a dose of 2 mg/kg every 48 hr for 12 consecutive days (22); 

Groups 3, 4, and 5 received various doses of carnosic acid (10, 20, and 40 mg/kg/day, respectively) via IP injection for a total of 16 days (20). These groups also received DOX at a dose of 2 mg/kg every 48 hr for 12 days, starting from day 4 of carnosic acid administration;

Group 6 received IP injections of vitamin E at a dose of 200 mg/kg every 48 hr for 16 days (23). These animals also received DOX at a dose of 2 mg/kg/48 hr for 12 days via IP injection, starting from day 4 of vitamin E administration;

Group 7 received carnosic acid at a dose of 40 mg/kg/day for a total of 16 days via IP injection. 

***Electrocardiography***

The animals were anesthetized the day after the final DOX administration. To induce sedation in the rats, an intraperitoneal injection of ketamine (100 mg/kg) and xylazine (10 mg/kg) was administered. A hot light bulb was employed to keep the room warm. Following that, to capture electrocardiogram (ECG) characteristics, four stainless steel needle electrodes were implanted subcutaneously into the right and left forepaws and hind paws of the animals. ECG recordings were made for each rat for ten minutes. The implanted electrodes were connected to a bioamplifier (Bio Amp ML136; AD Instruments, Australia) to record the ECG signals. The acquired data was then processed using LabChart 8.1.6 software, developed by AD Instruments. In the study, several ECG parameters were computed, including:

1. Heart rate (HR): The number of heartbeats per minute.

2. R-R interval (RRI): The interval between two consecutive R-waves on the ECG.

3. Q wave amplitude (QA): The voltage range from the deepest to the highest point of the Q wave on the ECG.

4. QT interval (QTI): The interval between the beginning of the Q wave and the end of the T wave on the ECG.

5. ST interval (STI): The interval between the end of the QRS complex and the start of the T wave on the ECG.

6. QRS duration: The time from the beginning of the Q wave to the end of the S wave on the ECG.

7. QRS complex amplitude (QRS voltage): The amplitude of the QRS complex on the ECG.

These parameters were calculated and analyzed to assess the cardiac function and electrical activity in the experimental animals (2).

***Hemodynamic study***

Heparin was given to the rats after their electrocardiograms were recorded to avoid blood clotting. Each animal had a longitudinal incision made in the neck region. This allowed the left carotid artery to be exposed. The artery was ligated distally and sutured proximally to prepare for hemodynamic monitoring. Following that, a small incision in the artery was created with mini-scissors, and a pressure transducer catheter (2F micromanometer-tipped) (SPR-407; Millar Instruments) was introduced to measure arterial systolic and diastolic blood pressure. The insertion of the catheter into the left ventricle (LV) was performed progressively, with continuous monitoring using the Powerlab system (AD Instruments, Australia) to capture hemodynamic measurements. Subsequently, several hemodynamic parameters were assessed, encompassing left ventricular diastolic pressure (LVDP), LV contractility (dP/dt), minimum rate of LV pressure increase (min dP/dt), and maximum rate of LV pressure increase (max dP/dt)(2). 

***Body weight and heart/body weight ratio***

Before conducting the hemodynamic investigation, the body weight of each rat was recorded. Once the surgical preparation was finished, the hearts of the rats were isolated, and their wet weights were measured. Subsequently, the heart weight to body weight ratio was calculated for each animal.

***Saving tissue samples***

After measuring the hemodynamic parameters, the rats were euthanized. The sternums were then opened to expose the heart, and all arteries and connective tissues were carefully removed. To eliminate blood, the heart was cleansed with a cold saline solution. The apex portion was preserved in formalin for subsequent histological analysis, while the remaining sections were rapidly frozen in liquid nitrogen and stored at -80 ^°^C for future processing.

***Histopathology***

The apex sample was removed for pathological analysis after the heart was separated. It was kept safe by soaking it in a neutral-buffered formalin solution. The isolated apex was subsequently subjected to histological processing, which included paraffin embedding, sectioning, and staining with trichrome dye. This staining protocol highlights muscle fibers red and collagen blue when viewed under a microscope. A pathologist with competence in cardiac histology evaluated the produced slides blindly. The degree of any abnormalities found across the experimental groups was graded using a semi-quantitative scoring system ranging from 1 to 4. The lowest score of 1 indicated no damage, while the highest score of 4 indicated the most serious changes.

***Assessing the amounts of MDA in isolated tissue***

MDA is a pivotal marker used to assess the extent of lipid peroxidation, and an increase in its concentration indicates an elevation in lipid peroxidation levels. In the presence of MDA and thiobarbituric acid (TBA) in an acidic medium, a pink color is generated, with the maximum absorption of color occurring at 532 nm. This color reaction serves as a characteristic indication of MDA presence.

To prepare a uniform solution, cardiac tissue was combined with 1.15% cold KCL. Subsequently, 0.5 ml of the homogenized tissue was mixed with 3 ml of 1% phosphoric acid and 1 ml of 0.6% TBA. The mixture was then subjected to boiling water for 45 min. Afterward, the tubes were cooled on ice, and 4 ml of n-butanol was added. The tubes were vigorously vortexed for one minute and then centrifuged at 3500 rpm for 10 min. The resulting supernatant was carefully transferred to a separate tube, and its absorbance was measured at 532 nm wavelengths. Finally, the MDA amounts were reported as nmol/g tissue (24, 25).

***Assessing the amounts of GSH in isolated tissue***

This technique stems from the reductive cleavage of 2-nitrobenzoic acid (DTNB) reagent with free sulfhydryl groups to produce a pale yellow color with the utmost absorption at 412 nm. Finally, GSH concentration was quantified as nmol/g of tissue (26). 

Heart tissue samples were homogenized in phosphate-buffered saline (PBS) at pH 7.4 to extract proteins. A volume of 500 µl of the homogenate was combined with an equal volume of 10% trichloroacetic acid. The resulting solution was centrifuged at 3000 x g for 10 min. The supernatant from the sample was then mixed with 2.5 ml of phosphate buffer at pH 8. Subsequently, 0.5 ml of DTNB reagent was added to initiate a colorimetric reaction. The absorbance of the mixture was measured at 412 nm using a spectrophotometer (27). 

***Western blot assay***

The Western blot method was employed to assess the levels of specific inflammatory and apoptotic markers in the heart tissues of rats belonging to different experimental groups. The heart tissues were lysed using a lysis buffer composed of Tris-HCl (50 mM, pH 7.4), ethylenediaminetetraacetic acid (EDTA)(2 mM), egtazic acid (EGTA)(2 mM), NaF (10 mM), sodium orthovanadate (Na_3_VO_4_)(1 mM), β-glycerophosphate (10 mM), complete protease inhibitor cocktail (Sigma Aldrich, USA), sodium deoxycholate (0.2% w/v), and phenylmethylsulfonyl fluoride (1 mM). The protein concentration of the samples was determined using the Bradford assay kit (Bio-Rad, USA) and adjusted accordingly. The resulting samples were mixed with a 2x sodium dodecyl sulfate (SDS) blue buffer, boiled for 5-7 min, aliquoted, and stored at -80 ^°^C (28). For the western blot analysis, the samples were loaded onto a 12% SDS-polyacrylamide gel and subjected to electrophoresis. Subsequently, the proteins were transferred to a polyvinylidene fluoride membrane (PVDF) (Bio-Rad, USA). The PVDF membranes were then blocked with 5% skim milk in Tris Buffered Saline with Tween® 20 (TBST) for two hours at room temperature. Following the blocking step, the PVDF membranes were washed three times with TBST (each time for five minutes) and incubated with specific antibodies for two hours at room temperature. The following antibodies were used in the western blot analysis:

1. Anti-TNF-α (Cell Signaling #3707, 1:1000)

2. Rabbit anti-IL-1β (Abcam #9722, 1:1000)

3. Rabbit polyclonal anti-Bax (Cell Signaling #2772, 1:1000)

4. Rabbit polyclonal anti-Bcl-2 (Cell Signaling #2870, 1:1000)

5. Rabbit monoclonal anti-caspase 3 (Cell Signaling #9665, 1:1000)

6. Rabbit monoclonal anti-caspase 8 (Cell Signaling #4790, 1:1000)

7. Rabbit monoclonal anti-caspase 9 (Cell Signaling #52873, 1:1000)

After incubation with the primary antibodies, the blots were washed three times with TBST. Subsequently, they were incubated with a rabbit horseradish peroxidase-conjugated anti-rabbit IgG secondary antibody (Cell Signaling #7074, 1:3000) for two hours. Chemiluminescence (Pierce, USA) was utilized to visualize the peroxidase-coated bands. An Alliance 4.7 Gel doc (UK) equipped with UVtec software (UK) was employed to measure the optical densities of the protein bands. Densitometric analysis of the protein bands was conducted using the UVtec software. Finally, the protein levels were normalized by comparing them to the reference bands of β-actin, which served as a control protein.

***Cell culture***

The MCF7 breast cancer cell line, which was initially derived from human breast carcinoma tissue, was acquired from the Pasteur Institute in Tehran, Iran. The cells were cultivated in RPMI 1640 medium supplemented with 100 μg/ml streptomycin, 100 U/ml penicillin, and 10% fetal bovine serum. The cell cultures were maintained in a humidified incubator at 37 ^°^C with 5% carbon dioxide. The MCF7 cells were passaged when the monolayer reached approximately 80% confluence to ensure logarithmic growth. Fresh medium was supplied to keep cells in an active proliferative state during experimentation (2).

***Cell viability***

The MCF7 cells were seeded at a density of 4000 cells per well. After 24 hr of seeding, the anticancer drug DOX was added to the cells at concentrations ranging from 1 to 10 μM. Following the DOX treatment, MTT reagent was added to each well at a final concentration of 0.5 mg/ml. The plates were then incubated for 24 hr, allowing the viable cells to metabolize the MTT into insoluble purple formazan crystals. After incubation, the culture media was removed, and each well was treated with 100-200 µl of DMSO solvent to dissolve the formazan crystals. The dissolved formazan produced a colored solution. Absorbance measurements were taken using a microplate reader at 545 nm, with a reference wavelength of 630 nm. These measurements provided information on the amount of formazan produced, which is indicative of cell viability and metabolic activity. The amount of formazan generated allowed for the measurement of live cells. The half-maximal inhibitory concentration (IC_50_) for DOX’s antiproliferative effects on MCF7 cells was obtained by evaluating absorbance values across multiple DOX concentrations tested over 24 hr.

Cells in the logarithmic growth phase were trypsinized, seeded into 96-well plates at a density of 4000 cells/well, and incubated to examine the effect of carnosic acid on the MCF7 cell line. After 24 hr, the cells were treated with various concentrations of carnosic acid (ranging from 1 to 10 μM). The MTT test was used to determine cell viability after 48 hr of incubation.

To check carnosic acid’s protective effects against DOX-induced cytotoxicity, MCF7 cells were first subjected to various doses of carnosic acid (ranging from 1 to 10 μM) that had no adverse effects on the cells. After 24 hr, a final concentration of 2.5 μM DOX (measured using the IC_50_ concentration assay) was added to the cell cultures. Cell survival was determined using the MTT test after another 24 hr of incubation. Cell viability was evaluated as a percentage of control cultures (1). 

***Statistics***

The statistical analysis was conducted using GraphPad Prism 6.0 software (GraphPad Prism Software Inc., San Diego, CA, USA). The data obtained from the experiments were presented as mean±standard deviation (Mean±SD). To compare the different experimental groups, a one-way analysis of variance (ANOVA) was performed, followed by the Tukey-Kramer test for *post hoc* analysis. Statistical significance was determined by *P*-values less than 0.05.

For the histopathological assessments, the data were reported as medians with interquartile ranges for each group. The statistical analysis of the histopathological data was performed using the Kruskal-Wallis nonparametric test, followed by Dunn’s Multiple Comparisons test for *post hoc* analysis.

## Results

***In vivo***

***DOX and carnosic acid effects on electrocardiographic recordings***

Table 1 presents the major variations in ECG recordings among the different experimental groups. In the control group, ECG features were typical. The administration of DOX (2 mg/kg every 48 hr, IP) for 12 days resulted in significant changes in several ECG parameters compared to the control group. These changes included a decrease in HR from 248 BPM in the control group to 178 BPM (*P*<0.01), as well as alterations in RRI, QA (*P*<0.001), STI (*P*<0.05), and QRS duration (*P*<0.001).

Pre-treatment with carnosic acid (10 mg/kg/day, IP) 4 days before starting DOX injection significantly improved HR (*P*<0.05), RRI (*P*<0.001), QA (*P*<0.01), and QRS duration (*P*<0.01) compared to the DOX group. Similarly, carnosic acid at a dose of 20 mg/kg improved HR (*P*<0.05) and decreased RRI (*P*<0.01), QA (*P*<0.001), and QRS duration (*P*<0.001) compared to the DOX group. However, carnosic acid at a dose of 40 mg/kg did not change the alterations induced by DOX in HR, RRI, and QA, but it did significantly decrease QRS duration (*P*<0.001).

No statistically significant differences were observed between the groups receiving 10 or 20 mg/kg carnosic acid treatment and the control group for the evaluated parameters. However, when compared to the control group, the combination treatment with 40 mg/kg carnosic acid and DOX showed significant differences in HR, RRI (*P*<0.01), and QA (*P*<0.05).

Additionally, administration of vitamin E was found to significantly increase heart rate (*P*<0.05) and decrease RRI, QA (*P*<0.01), and QRS duration (*P*<0.001) compared to the DOX group. No statistically significant differences were found between the vitamin E treatment group and the control group for any of the analyzed ECG parameters. Similarly, no significant differences were detected between the control group and the group receiving 40 mg/kg carnosic acid alone for the evaluated ECG measures.

Although DOX administration was found to affect QT and increase QRS voltage, these variations were not statistically significant across the studied groups.

***DOX and carnosic acid effects on arterial (carotid) blood pressure***

In Table 2, the changes in systolic pressure, diastolic pressure, and MAP are presented. The DOX group exhibited a significant decrease in systolic pressure (from 129 mmHg in the control group to 96 mmHg), diastolic pressure (from 99 mmHg in the control group to 60 mmHg), and MAP (from 109 mmHg in the control group to 72 mmHg) compared to the control group (*P*<0.01).

Among the treatment groups, only administration of carnosic acid at a dose of 20 mg/kg was able to improve the DOX-induced alterations in systolic pressure (*P*<0.01) and MAP (*P*<0.05) compared to the DOX group (Table 2). However, co-administration of carnosic acid at doses of 10 mg/kg and 40 mg/kg with DOX did not have a significant effect on systolic pressure, diastolic pressure, or MAP compared to the DOX group.

When compared to the control group, the combination treatment of 10 mg/kg carnosic acid with DOX showed statistically significant differences in systolic blood pressure, diastolic blood pressure, and MAP (*P*<0.01). Similarly, the group receiving 40 mg/kg carnosic acid along with DOX exhibited highly significant differences compared to the control group in systolic pressure, diastolic pressure, and MAP (*P*<0.001).

Vitamin E supplementation did not impact the alterations in hemodynamic indices produced by DOX when compared to animals receiving DOX alone. The vitamin E combined with the DOX group exhibited statistically significant differences versus controls for systolic pressure, diastolic pressure, and MAP (*P*<0.05). No significant differences were detected in any of the hemodynamic measurements, including systolic pressure, diastolic pressure, and MAP, between the control rats and those treated exclusively with 40 mg/kg carnosic acid.

***DOX and carnosic acid effects on the function of left ventricular***

The Dox group exhibited decreased values of max dP/dt, min dP/dt, and LVDP compared to the control group, with statistical significance (*P*<0.05). The administration of carnosic acid at the dose of 10 mg/kg did not have a noticeable impact on these factors. However, at the dose of 20 mg/kg, carnosic acid significantly improved max dP/dt, min dP/dt (*P*<0.001), and LVDP (*P*<0.05). Furthermore, at a dose of 40 mg/kg, carnosic acid was able to mitigate the negative effects of Dox on max dP/dt and min dP/dt (*P*<0.05). Significant differences were observed in max dP/dt (*P*<0.01), min dP/dt (*P*<0.05), and LVDP (*P*<0.001) between the carnosic acid (10 mg/kg) plus DOX group and the control group. Similarly, significant differences were found in max dP/dt (*P*<0.05) and min dP/dt (*P*<0.01) between the carnosic acid (20 mg/kg) plus DOX group and the control group. Moreover, a significant improvement in LVDP (*P*<0.05) was observed between the carnosic acid (40 mg/kg) plus DOX group and the control group.

The administration of vitamin E did not have a significant effect on max dP/dt, min dP/dt, and LVDP. However, a statistically significant difference was observed in LVDP between the vitamin E plus DOX group and the control group (*P*<0.001). When comparing the control group to the group receiving only 40 mg/kg carnosic acid treatment, no statistically significant differences were found for any of the measured hemodynamic parameters (Table 3).

***Carnosic acid effect on body, heart, and heart/body weight ratio***

Receiving DOX (2 mg/kg every 48 hr, IP) for a duration of 12 days did not affect body weight, but it led to a significant increase in heart weight (*P*<0.05) and heart-to-body weight ratio (*P*<0.01) compared to the control group. However, pretreatment with carnosic acid at doses of 10, 20, and 40 mg/kg/day (IP) for 4 days prior to initiating DOX administration resulted in a notable decrease in heart weight when compared to the DOX group (*P*<0.01). Furthermore, carnosic acid at doses of 10 mg/kg (*P*<0.01), and 20 and 40 mg/kg (*P*<0.001) exhibited the ability to reduce the heart-to-body weight ratio compared to the DOX group (Table 4). No statistically significant differences were observed between the groups treated with 10, 20, or 40 mg/kg carnosic acid and the control group in terms of heart weight, body weight, or the heart-to-body weight ratio.

While the administration of vitamin E did not have a direct impact on heart or body weights, it significantly decreased the heart-to-body weight ratio compared to animals treated with DOX (*P*<0.001). However, no statistically significant differences were found between the vitamin E group and the control group. Additionally, the carnosic acid 40 mg/kg treatment group displayed no meaningful differences versus controls for heart weight, body weight, or their ratio.

***Carnosic acid effect on cardiac histopathological changes***

**Figure 1:**
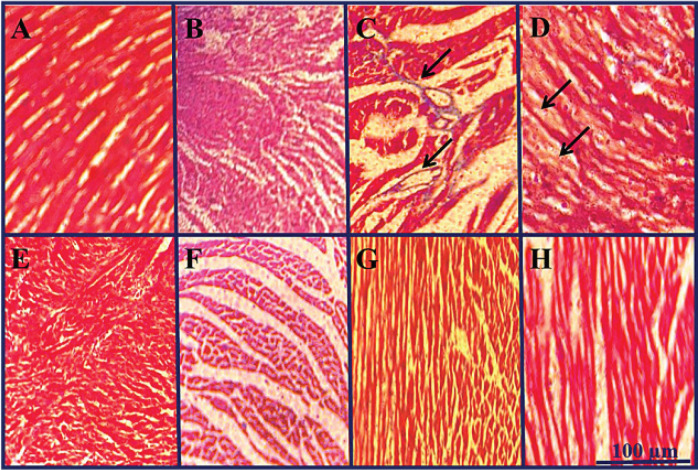
Effect of carnosic acid on doxorubicin induced histopathological changes in the heart tissue of control, doxorubicin, carnosic acid, and vitamin E treated groups

**Figure 2:**
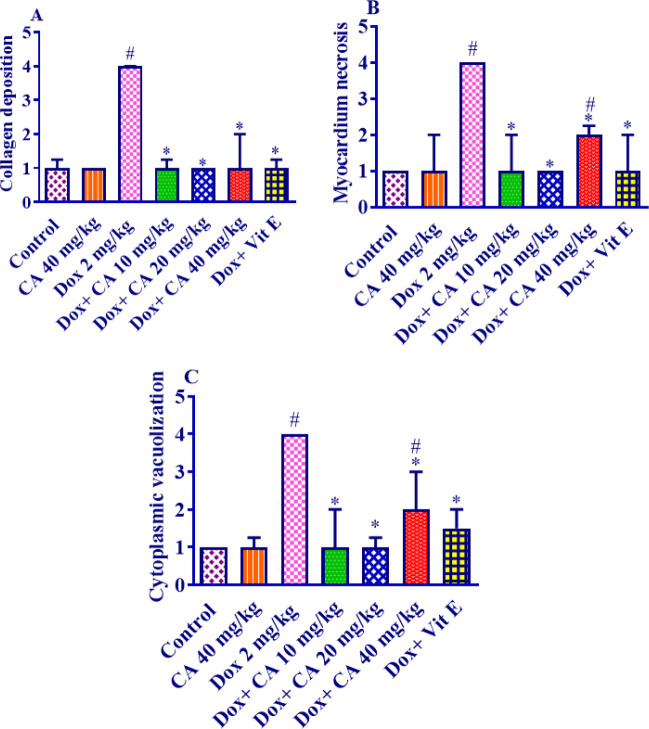
Effect of carnosic acid and doxorubicin on A) Collagen deposition, B) myocardial necrosis, and C) Cytoplasmic vacuolization in cardiac tissue of control, doxorubicin, carnosic acid, and vitamin E treated groups (on a scale of 1 to 4, with 1 signifying normal and 4 indicating the most serious harm)

Figures 1 A and B show the normal cardiac tissue in the control group and samples that received carnosic acid (40 mg/kg), respectively. DOX prescription caused histopathological alterations in heart tissue including extensive fibrosis and collagen deposition (Figure 1C), scattered and necrotic cardiomyocytes, degeneration of myocardial fibers, and cytoplasmic vacuolization (Figure 1D). These variations were not realized in other treatment groups (Figure 1 E-H). The administration of DOX led to a notable increase in collagen deposition, myocardial necrosis, and cytoplasmic vacuolization when compared to the control group, with statistical significance (*P*<0.001). The concurrent administration of carnosic acid (at doses of 10, 20, and 40 mg/kg) or vitamin E with DOX resulted in a remarkable reduction in collagen deposition compared to the DOX group, with statistical significance (*P*<0.001) (Figure 2). When carnosic acid at a dose of 40 mg/kg was prescribed alongside DOX, significant alterations were observed in myocardial necrosis (*P*<0.05) and cytoplasmic vacuolization (*P*<0.01) compared to the control group. However, administration of carnosic acid at doses of 10 mg/kg, 20 mg/kg, or vitamin E concurrent with DOX, as well as carnosic acid at a dose of 40 mg/kg alone, did not result in any significant changes compared to the control group.

***Carnosic acid effect on lipid peroxidation***

Administration of DOX (2 mg/kg every 48 hr, IP) for a period of 12 days resulted in a significant increase in the MDA level in the heart tissue compared to the control group, demonstrating statistical significance (*P*<0.001). However, when carnosic acid supplementation at doses of 10, 20, and 40 mg/kg/day (IP) was initiated 4 days before DOX administration, there was a remarkable decrease in the MDA level compared to the control group (*P*<0.001). No statistically significant differences were observed in the MDA levels among any of the treatment groups when compared to the control group.

**Figure 3:**
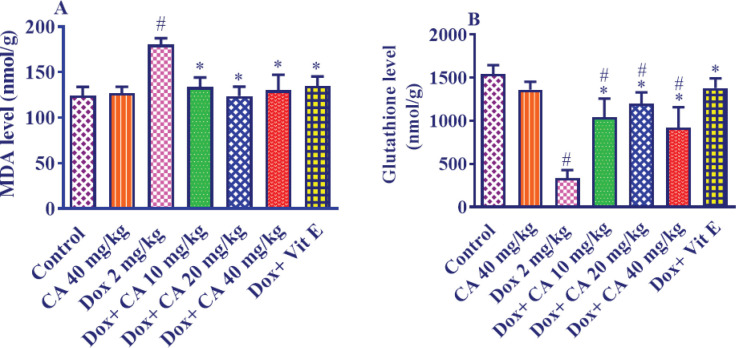
Effect of carnosic acid on A) lipid peroxidation and B) GSH reduction induced by DOX in the heart tissue of control, doxorubicin, carnosic acid, and vitamin E treated groups

In contrast, pretreatment with vitamin E at a dosage of 200 mg/kg every other day for four days before DOX administration significantly reduced MDA levels in comparison to the group receiving only DOX (*P*<0.001). The combination of vitamin E and DOX did not result in significant changes in MDA levels when compared to the control group. Furthermore, no significant differences in MDA levels were observed between the group treated with carnosic acid at a dose of 40 mg/kg and the control group (Figure 3A).

***Carnosic acid effect on GSH reduction***

The administration of DOX at a dose of 2 mg/kg every 48 hr for a duration of 12 days led to a significant decrease in the GSH level in the heart tissue compared to the control group, with statistical significance (*P*<0.001). However, when carnosic acid was administered at doses of 10, 20, and 40 mg/kg/day via the intraperitoneal route for four days before DOX administration, there was a significant increase in the GSH content in the heart tissue compared to the DOX group (*P*<0.001). Statistically significant differences in GSH content were observed between the control group and the groups receiving combinations of carnosic acid at doses of 10 mg/kg (*P*<0.001), 20 mg/kg (*P*<0.01), and 40 mg/kg (*P*<0.001) in conjunction with DOX.

Furthermore, in comparison to the rats receiving only DOX, pre-treatment with vitamin E at a dosage of 200 mg/kg intraperitoneally every other day significantly elevated the GSH levels compared to the DOX alone group (*P*<0.001). However, no statistically significant differences in GSH levels were found between the group receiving vitamin E plus DOX and the control group. Additionally, the analysis indicated no significant difference in GSH content between the group treated with carnosic acid at a dose of 40 mg/kg alone and the control group (Figure 3B).

***Carnosic acid effect on the Bax/Bcl-2 ratio***

The western blot analysis was conducted on the combination of DOX and carnosic acid at a dose of 20 mg/kg, which was determined to be the most effective dosage. 

**Figure 4:**
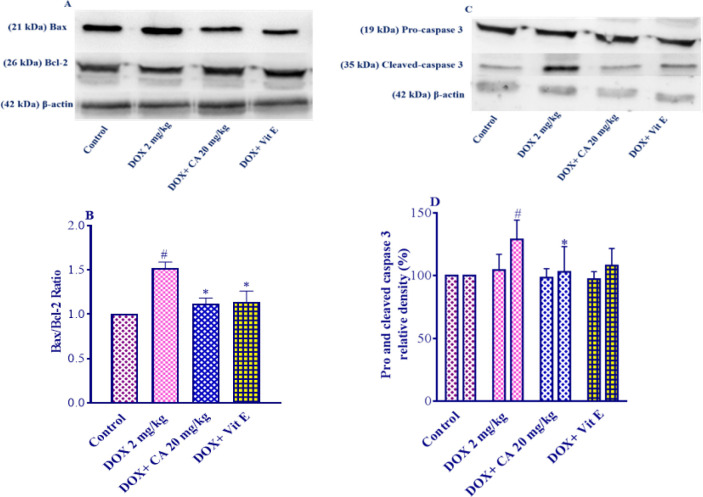
Effect of carnosic acid on the Bax/Bcl-2 ratio and pro and cleaved-caspase3 levels in the heart tissue of control, doxorubicin, carnosic acid, and vitamin E treated groups

Following the administration of DOX, there was a significant increase in the Bax/Bcl-2 ratio in the heart tissue (*P*<0.01). However, when carnosic acid supplementation at a dose of 20 mg/kg was combined with DOX, there was a notable reduction in the Bax/Bcl-2 ratio (*P*<0.05) compared to the group treated with DOX alone (Figures 4A and B). Similarly, the co-administration of vitamin E with DOX also resulted in a decrease in the Bax/Bcl-2 ratio compared to the DOX group (*P*<0.05). No substantial variations were detected in the carnosic acid plus DOX and vitamin E plus DOX groups versus the control group.

***Carnosic acid effect on pro and cleaved-caspase 3 levels***

The levels of cleaved caspase-3 in the heart tissue were significantly increased following DOX administration (*P*<0.05). However, when carnosic acid supplementation at a dose of 20 mg/kg was combined with DOX, there was a notable decrease in the levels of cleaved caspase-3 (*P*<0.05) compared to the group treated with DOX alone (Figures 4C and D). In contrast, the reduction of cleaved-caspase 3 levels by vitamin E was not statistically significant versus the DOX group. There were no statistically noteworthy alterations observed between the carnosic acid plus DOX and vitamin E plus DOX groups and the control group. 

The analysis found no statistically significant differences in pro-caspase 3 levels across any of the experimental treatment groups.

***Carnosic acid effect on pro and cleaved-caspase 8 levels***

**Figure 5:**
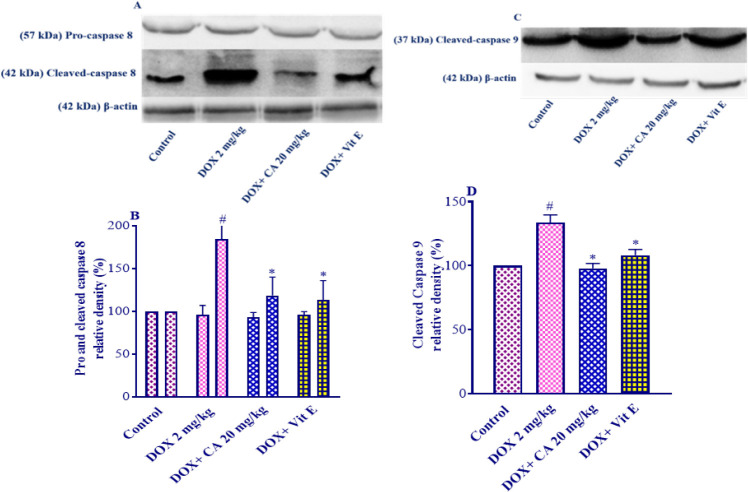
Effect of carnosic acid on pro and cleaved-caspase8 and caspase-9 levels in the heart tissue of control, doxorubicin, carnosic acid, and vitamin E treated groups

The administration of DOX resulted in a significant increase in the levels of cleaved caspase-8 in cardiac tissue (*P*<0.001). However, when carnosic acid supplementation at a dose of 20 mg/kg was combined with DOX, there was a significant decrease in the levels of cleaved caspase-8 (*P*<0.001) compared to the group treated with DOX alone (Figures 5A and B). Additionally, the co-administration of vitamin E also led to a significant reduction in the levels of cleaved caspase-8 compared to the DOX group (*P*<0.001). It is noteworthy that neither the carnosic acid plus DOX group nor the vitamin E plus DOX group showed any statistically significant differences compared to the control group.

Furthermore, there were no statistically significant changes observed in the levels of pro-caspase-8 when comparing all the experimental groups.

***Carnosic acid effect on the cleaved-caspase 9 level***

DOX administration resulted in a significant increase in the levels of cleaved caspase-9 in the heart tissue compared to the control group (*P*<0.001). However, when carnosic acid supplementation at a dose of 20 mg/kg was combined with DOX, there was a significant reduction in the levels of cleaved caspase-9 compared to the DOX group (*P*<0.001). Similarly, co-administration of vitamin E also significantly decreased the levels of cleaved caspase-9 compared to the DOX group (*P*<0.01) (Figures 5C and D).

Notably, no statistically significant differences were found in the levels of pro-caspase-9 between the control group and both carnosic acid plus DOX treatment groups or the vitamin E plus DOX group.

***Carnosic acid effect on IL-1β level***

**Figure 6:**
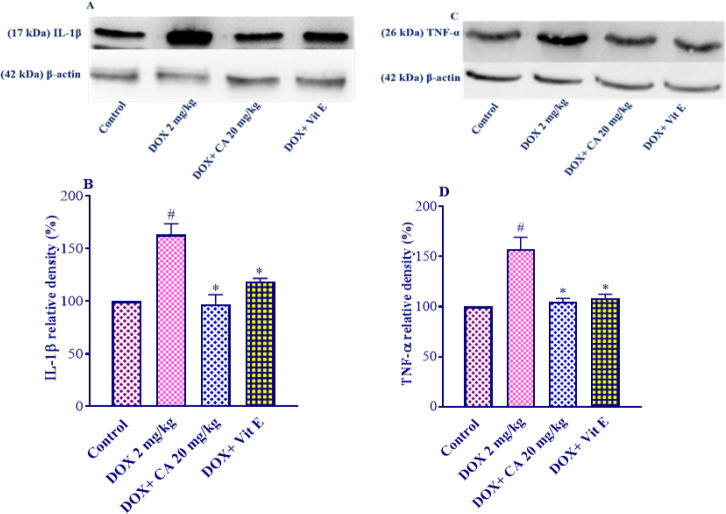
Effect of carnosic acid on IL-1β and TNF-α levels in the heart tissue of control, doxorubicin, carnosic acid, and vitamin E treated groups

DOX administration led to a significant increase in the levels of IL-1β in cardiac tissue compared to the control group (*P*<0.001). However, when carnosic acid supplementation at a dose of 20 mg/kg was combined with DOX, there was a significant reduction in the levels of IL-1β compared to the DOX group (*P*<0.001). Similarly, supplementation of vitamin E considerably reduced the IL-1β levels versus the DOX group (*P*<0.01) (Figures 6A and B). The carnosic acid plus DOX and vitamin E plus DOX groups did not exhibit any statistically significant differences versus controls.

***Carnosic acid effect on TNF-α level***

Following DOX administration, there was a significant increase in the levels of TNF-α in the heart tissue compared to the control group (*P*<0.001). However, when carnosic acid supplementation at a dose of 20 mg/kg was combined with DOX, there was a significant reduction in the levels of TNF-α compared to the DOX group (*P*<0.001) (Figures 6C and D). Similarly, the supplementation of vitamin E with DOX also resulted in a significant decrease in the levels of TNF-α compared to the DOX group (*P*<0.001). Importantly, there were no significant differences observed among the carnosic acid plus DOX treatment groups, the vitamin E plus DOX group, and the control group.

***In vitro***

***DOX effect on MCF7 cells viability***

**Figure 7:**
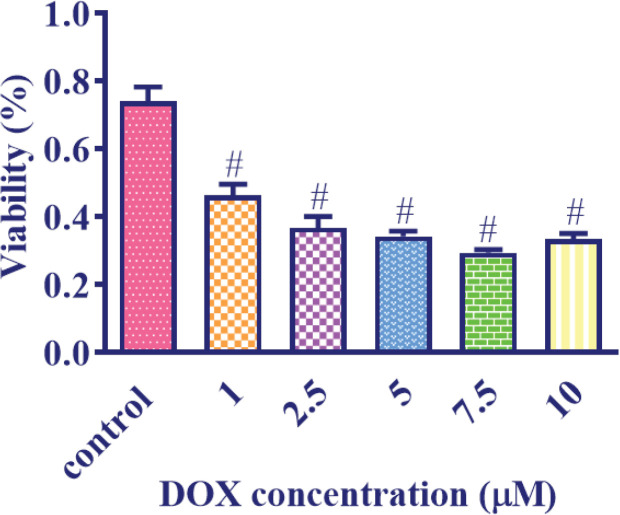
Effect of 1-10 μM doxorubicin (DOX) concentrations on MCF7 cells for 24 hr

MCF7 cells were subjected to different concentrations of DOX ranging from 1 μM to 10 μM for a duration of 24 hr. Subsequently, a cell viability assay using the MTT method was conducted to assess the impact of DOX on cell viability. By utilizing data analysis software, specifically GraphPad Prism 6, the IC_50_ (half-maximal inhibitory concentration) of DOX was determined to be 2 μM (Figure 7).

***Carnosic acid effect on MCF7 cells viability***

**Figure 8:**
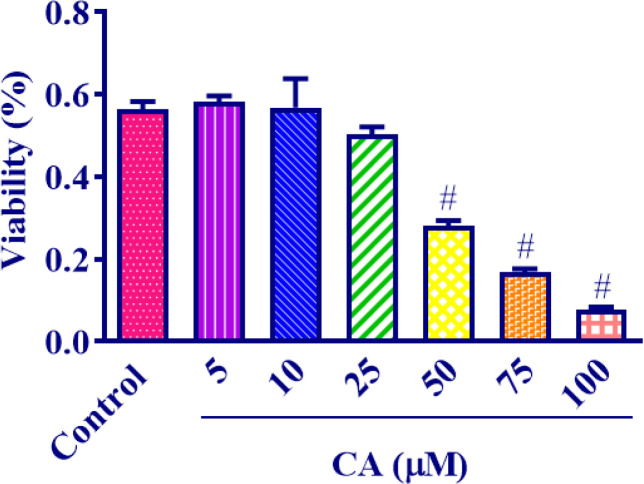
Effect of 5-100 μM carnosic acid concentrations on the viability

In order to evaluate the impact of carnosic acid on the viability of MCF7 cells, different concentrations of carnosic acid ranging from 5 μM to 100 μM were administered to the cells for a period of 24 hr. The findings revealed that carnosic acid exhibited a significant reduction in MCF7 cell viability at concentrations exceeding 50 μM. Analysis of the data using appropriate methods determined the IC_50_ of carnosic acid to be 49.28 μM (Figure 8).

***Carnosic acid effect on the cytotoxicity of MCF7 cells induced by DOX***

**Figure 9:**
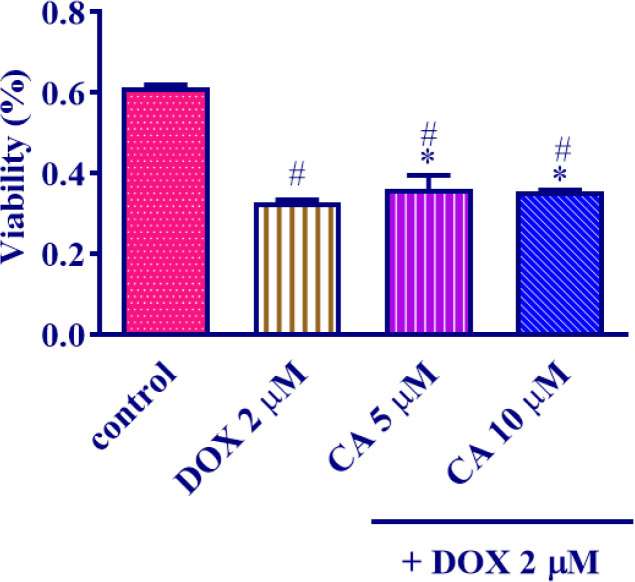
Effect of 5 and 10 μM carnosic acid concentrations on the viability

To explore the potential protective effects of carnosic acid against DOX-induced toxicity, MCF7 cells were subjected to carnosic acid treatments at concentrations of 5 μM and 10 μM for a duration of 24 hr. Following this, DOX at a concentration of 2 μM was introduced to the wells. On the subsequent day, cell viability was assessed utilizing an MTT assay. Figure 9 illustrates the results, demonstrating that both 5 μM and 10 μM concentrations of carnosic acid significantly increased cell viability in comparison to the group treated with DOX alone (*P*<0.001 for 5 μM; *P*<0.01 for 10 μM). Moreover, both the 5 μM (*P*<0.001) and 10 μM (*P*<0.001) carnosic acid treatments exhibited statistically significant differences when compared to the untreated control group.

## Discussion

The study aimed to assess the protective properties of carnosic acid against DOX-induced cardiotoxicity in rat hearts and its cytotoxic effects on MCF7 cell lines, which are used to study the growth inhibition effects of chemotherapy drugs. The experiment involved administering DOX (2 mg/kg, IP) every 48 hr for 12 days to induce cardiotoxicity in rats. Various parameters were evaluated, including electrocardiogram parameters (RRI, HR, QA, QRS duration, and STI), arterial (carotid) pressure, hemodynamic parameters (LVDP, Min dP/dt, and Max dP/dt), heart weight, heart weight-to-body weight ratio, levels of MDA and GSH (oxidative stress markers), apoptotic and anti-apoptotic factors (Bax, Bcl-2, caspase 3, 8, 9), inflammatory markers (IL1-β, TNF-α), and histopathological characteristics of the rat hearts. The administration of DOX resulted in significant alterations in these parameters, indicating cardiotoxicity. However, treatment with carnosic acid at doses of 10, 20, and 40 mg/kg for 16 days (starting 4 days before DOX injection) ameliorated these changes, suggesting a protective effect. Additionally, vitamin E, used as a reference drug, was able to alleviate some of the adverse effects caused by DOX, including changes in HR, RRI, QA, QRS duration, MDA, GSH, caspase 8, 9, IL1-β, and TNF-α levels in the rat hearts. In the *in vitro* part of the study using MCF7 cell lines, DOX was found to suppress cell viability, indicating its cytotoxic effect as a chemotherapy drug. However, carnosic acid at concentrations of 5 and 10 μM did not affect the cytotoxic effect of DOX on cell growth, suggesting that carnosic acid did not interfere with DOX’s cytotoxic properties as a chemotherapy drug.

DOX is extensively used to control and manage multiple cancers in humans. However, its prescription is restricted because of the various side effects. One of the most common toxic results of patients receiving DOX is cardiomyopathy (2). Congestive heart failure was reported in approximately 1/3 of patients who take an accumulative dose of DOX (more than 550 mg/m^2^)(29). Moreover, some other studies claim that cardiomyopathy might develop even at much lower cumulative doses than thought earlier, but it may manifest years after treatment and exposure to DOX, particularly in pediatric oncology patients (30). The animal model utilized in this study was previously described (7) and described by damages comparable to what was stated by other research projects (31). 

***In vivo***

***DOX and carnosic acid effects on ECG***

It has been reported that administration of DOX might cause several alterations in electrocardiogram parameters, including left axis deviation (1, 2), elongation or shortening of ST segment, T-wave inversion, extended QTI, declined QRS voltage (32), increased RRI, and QA (7, 22). Our results also reinforce the previous studies since it was observed that DOX injection caused a significant decrease in HR and elevated RRI, QA, STI, and QRS duration. However, the administration of carnosic acid pointedly reversed the alterations of HR, RRI, QA, and QRS duration. In contrast to our results, Colak and colleagues (33) reported that DOX caused an elevation in HR. This difference might be explained by the rats they used (female rats) and the interference of estrogen and progesterone hormones. Furthermore, the researchers employed a single dosage of DOX at 20 mg/kg in their experimental protocol. The probable underlying mechanisms for these changes might be reversible myocardial edema and the functional alterations in the adrenergic system that were triggered by DOX. Also, HR variability might be related to the weakened autonomic control of the heart (34). Another probability is the down-regulation of adrenergic beta-receptors stated to take place in the hearts of rats that received DOX (35) and the subsequent narrowed reactivity of the heart to the regular sympathetic stimulations. It was also reported that the DOX-induced elongation of action potential duration, alterations in the restoration period of the transmembrane action potential, and changes in Ca^2+^ movements through the cellular membrane might be responsible for the observed ECG fluctuations (36). To date, there have been no studies specifically examining the effects of carnosic acid on electrocardiographic recordings. However, the cardioprotective properties of carnosic acid may stem from its known anti-oxidant, anti-inflammatory, and anti-apoptotic effects, which will be elaborated upon in the subsequent paragraphs (37, 38).

***DOX and carnosic acid effects on hemodynamic factors***

The data of the hemodynamic recordings exhibited myocardial damage formed by DOX, as indicated by the decrease in systolic and diastolic pressure and MAP as well. Furthermore, DOX suppressed the left ventricular Max dP/dt, Min dP/dt, and LVDP. However, carnosic acid demonstrated its cardioprotective property in contrast to DOX-induced cardiac impairments by inverting DOX alterations on the systolic pressure, MAP, Max dP/dt, Min dP/dt, and LVDP. This finding aligns with a study conducted by Razmaraii *et al*. (7, 22), which also reported that DOX prescription caused a significant reduction in systolic and diastolic pressure, MAP, Max dP/dt, and Min dP/dt. However, no significant difference in QT interval was observed among their study groups. These alterations might be because of the attenuated expression of the contractile proteins that are related to myofibrillar damage and decreased myocardial contractility. Likewise, a reduction in sarcoplasmic reticular ATPase may be followed by myocardial diastolic abnormal function (39). It has also been stated that calcium homeostasis in the myocardium can be affected by receiving DOX (40). It is also essential to note that the alterations observed in blood pressure after the prescription of DOX might be due to the nephrotoxic property of this medicine (41). So far as we know, the effects of carnosic acid on hemodynamic parameters have not been thoroughly examined in previous research. However, the cardioprotective properties of carnosic acid are based on its anti-oxidant, anti-inflammatory, and anti-apoptotic actions (37, 38), which are discussed in the following sections.

***DOX and carnosic acid effects on body, heart, and heart/body weight ratio***

Upon examining various cardiac metrics, DOX treatment resulted in higher heart weight and increased heart/body weight proportion. However, administering carnosic acid mitigated these changes induced by DOX. Consistent with our findings, prior research also found no difference in body weights of rats before and after DOX exposure, though their heart weights were significantly greater than controls (33, 42). A study in mice found that carnosic acid directly decreased the heightened heart-to-body weight ratio seen in the isoproterenol group, highlighting carnosic acid’s protective effects on these cardiac parameters altered by problematic stimulant medications (19).

***DOX and carnosic acid effects on cardiac histopathological alterations***

As part of this study, histopathological investigations displayed that the administration of DOX led to necrotic and scattered cardiomyocytes, as well as wide fibrosis. The rearrangements were recovered by carnosic acid treatment. In agreement with our findings, it was outlined that treating with DOX-induced usual signs of cardiotoxicity such as inflammation, loss of cross striation, vacuolization of cardiomyocytes, apoptosis, and focal necrosis (43). A recent investigation also stated that DOX administration resulted in considerable collagen fiber deposition as exhibited by an augmented percentage of interstitial fibrosis area in heart tissue sections in mice (44). The results of other investigations indicated that carnosic acid could remarkably ameliorate cardiac histological deformities in isoproterenol-injected rats (37). Moreover, it has been illustrated that carnosic acid attenuated DOX-induced associated pathological consequences in rats’ cardiac tissue (45).

***DOX and carnosic acid effects on oxidative stress of cardiac tissue***

One of the main proposed mechanisms of cardiotoxicity induced by DOX is the amplified oxidative stress, verified by elevated amounts of reactive oxygen species and lipid peroxidation (46, 47). Zhang *et al.* claimed that following DOX administration to mice a significant elevation was seen in the amount of MDA and protein carbonyl in cardiac tissue, but the GSH level was reduced (44). The perceived GSH deficiency related to DOX administration might be attributable to GSH depletion in the collaborations with DOX-induced free radicals with bio-membrane and the consequent lipid peroxidation (48). In our study, we observed that DOX prescription triggered increased MDA levels and reduced GSH levels, but carnosic acid reversed MDA and GSH amounts. In accordance with our research, it was shown that carnosic acid displayed a potent cardioprotective influence against isoproterenol-induced myocardial damage in mice by preserving the status of myocardial anti-oxidants and extending endogenous anti-oxidants (19). Also, receiving carnosic acid developed GSH levels and lowered MDA levels in the liver of rats suffering from hepatotoxicity stimulated by acetaminophen (49).

***DOX and carnosic acid effects on the levels of inflammatory markers in cardiac tissue***

A prior study detailed how DOX can trigger a pathogenic inflammatory response in cardiac tissues by augmenting NF-κB and promoting the generation of downstream pro-inflammatory cytokines (50). NF-κB is a transcription agent that moderates the host inflammatory reactions and adjusts the expression of pro-inflammatory mediators including nitric oxide (NO), cyclooxygenase-2 (COX-2), IL-18, IL-1β, IL-6, and TNF-α (51). Consistently, it was explained that DOX outstandingly elevated p-NF-κB, IL-18, TNF-α, IL-1β, NO, IL-6, and COX-2 expressions (44). Our findings revealed that IL-1β and TNF-α amounts were increased notably in the DOX group, meanwhile, carnosic acid administration reduced the amount of these inflammatory markers. Recent research reported that carnosic acid protects against lipopolysaccharide-induced severe lung injury by suppressing the expression of IL-6, TNF-α, IL-1β, and NF-κB in mice (52). 

***DOX and carnosic acid effects on apoptosis in cardiac tissue***

There is some evidence that DOX induces apoptosis in cardiomyocytes. The development of hydrogen superoxide and peroxide is associated with DOX-induced cardiomyocyte toxicity (53). Apoptosis is controlled by a chain of regulatory proteins. The members of the Bcl-2 family were observed to be the major regulators of apoptosis (54). This family includes proapoptotic (including Bax) and anti-apoptotic (such as Bcl-2) proteins (55). It was pointed out that DOX induced noticeable apoptosis in cardiomyocytes through up-regulating caspase-3 activity, increasing Bax expression, and reducing the Bcl-2 amount (56). In the present study, receiving DOX caused a significant elevation in Bax, cleaved caspase 3, 8, and 9 amounts, but it had no effect on the Bcl-2 and pro-caspase 3, 8 levels. Carnosic acid pretreatment expressively prohibited the DOX-induced apoptosis in the heart tissue, which explains the histopathological alteration and antioxidative enzyme activities. In agreement with our results, it was claimed that carnosic acid administration decreased myocardial expression of Bax, cleaved caspase-3, 9, and restored Bcl-2 amount in comparison with the isoproterenol group (19). Treating rats with subarachnoid hemorrhage triggered by brain injury with carnosic acid boosted the expression of Bcl-2 decreased the expression of Bax and cleaved caspase-3 (57).

Overall, based on our findings, the different concentrations of carnosic acid used in this study were beneficial in ameliorating DOX-induced cardiotoxicity but among all carnosic acid doses, a dose of 20 mg/kg was more advantageous. It is possible to conclude that lower (<10 mg/kg) doses of carnosic acid might not be strong enough to ameliorate the alterations caused by DOX in the heart tissue and higher doses (>40 mg/kg) of carnosic acid may disclose toxic effects.

In contrast to carnosic acid, vitamin E pretreatment was unable to prevent the changes in carotid pressures, minimum dP/dt, LVDP, and MAP induced by DOX administration. These results support previous findings that vitamin E supplementation did not impact heart failure risk in healthy women (58), suggesting its anti-oxidant properties alone may not be enough to treat cardiotoxicity induced by DOX. Therefore, it can be hypothesized that additional mechanisms beyond vitamin E anti-oxidant effects are required to protect against DOX-induced cardiac damage.

***In vitro***

Additionally, our team assessed the effect of carnosic acid co-administration along with DOX on MCF7 cells to make sure that carnosic acid would not reduce the cytotoxic effect of DOX as a chemopreventive agent, and the results of the MTT assay demonstrated that DOX showed a cytotoxic influence on MCF7 cells, which was in agreement with earlier investigations (59), and carnosic acid (5, 10 µM) treatment along with DOX had no significant impact on DOX-induced cell toxicity on MCF7 cells. 

**Table 1:** Doxorubicin and carnosic acid effects on electrocardiogram pattern and changes (recorded from limb lead II) in control, doxorubicin, carnosic acid, and vitamin E treated groups

Groups	HR (BPM)	RRI (s)	QA (58)	QT (s)	STI (s)	QRS duration (s)	QRS voltage (58)
Control	248±5.84	0.24±0.00	0.061±0.00	0.051±0.00	0.016±0.00	0.038±0.00	0.47±0.09
CA (40 mg/kg)	213.33±15.04	0.27±0.01	0.083±0.00	0.052±0.00	0.016±0.00	0.037±0.00	0.46±0.08
DOX (2 mg/kg)	178.66±15.51 #	0.35±0.02 #	0.12±0.00 #	0.048±0.00	0.020±0.00 #	0.049±0.00 #	0.68±0.03
DOX + CA (10 mg/kg)	240.33±8.61 *	0.24±0.01 *	0.08±0.00 *	0.051±0.00	0.018±0.00	0.041±0.00 *	0.66±0.05
DOX + CA (20 mg/kg)	244.33±20.47 *	0.25±0.02 *	0.075±0.00 *	0.052±0.00	0.018±0.00	0.039±0.00 *	0.54±0.01
DOX + CA (40 mg/kg)	174.66±7.37 #	0.35±0.01 #	0.093±0.00 #	0.054±0.00	0.017±0.00	0.040±0.00 *	0.76±0.06
DOX + Vit E (200 mg/kg)	242±8.85 *	0.24±0.01 *	0.08±0.00 *	0.050±0.00	0.018±0.00	0.040±0.00 *	0.61±0.03

The data were analyzed using ANOVA followed by Tukey’s *post-hoc* test. Each value represents the mean±SEM, with n=6 animals per group, # compared to the control group; * compared to the DOX group

DOX: doxorubicin, CA: carnosic acid, Vit E: vitamin E, HR: heart rate; RRI: RR interval; QA: Q amplitude; STI: ST interval

**Table 2:** Doxorubicin and carnosic acid effects on arterial blood pressure in control, doxorubicin, carnosic acid, and vitamin E treated groups

Groups	Systolic pressure (mmHg)	Diastolic pressure (mmHg)	MAP (mmHg)
Control	129.66±2.35	99.33±3.77	109.44±3.28
CA (40 mg/kg)	130.5±5.75	89.66±3.50	103.27±3.94
DOX (2 mg/kg)	96.33±5 #	60.5±4.86 #	72.44±4.90 #
DOX + CA (10 mg/kg)	88.83±6.78 #	63.16±8.72 #	71.72±7.83 #
DOX + CA (20 mg/kg)	130.33±3.99 *	86.5±4.27	101±3.82 *
DOX + CA (40 mg/kg)	90.5±9.13 #	56.66±8.65 #	67.94±8.80 #
DOX + Vit E (200 mg/kg)	101.16±8.25 #	69.33±6.87 #	79.94±7.32 #

The data were analyzed using ANOVA followed by Tukey’s *post-hoc* test. Each value represents the mean±SEM, with n=6 animals per group

^#^ compared to the control group; ^*^ compared to the DOX group

DOX: doxorubicin, CA: carnosic acid, Vit E: vitamin E, MAP: mean arterial pressure

**Table 3:** Doxorubicin and carnosic acid effects on cardiac hemodynamic function in control, doxorubicin, carnosic acid, and vitamin E treated groups

Groups	Max dP/dt (mmHg/s)	Min dP/dt (mmHg/s)	LVDP (mmHg)
Control	6634.00±327.12	-4785.00±306.55	130.36±8.15
CA (40 mg/kg)	6277.83±568.69	-4729.66±558.39	109.52±10.65
DOX (2 mg/kg)	3564.33±813.03 #	-2426.83±417.67 #	93.05±7.63 #
DOX + CA (10 mg/kg)	2998.16±844.08 #	-2188.33±437.6 #	69.98±8.65 #
DOX + CA (20 mg/kg)	10025.67±733.74 * #	-7538.00±660.73 * #	131.52±7.57 *
DOX + CA (40 mg/kg)	6480.66±767.02 *	-4991.33±708.68 *	93.32±3.99 #
DOX + Vit E (200 mg/kg)	4339.16±674.86	-4145.16±329.54	80.3±4.51 #

The data were analyzed using ANOVA followed by Tukey’s *post-hoc* test. Each value represents the mean±SEM, with n=6 animals per group

^#^ compared to the control group; ^*^ compared to the DOX group

DOX: doxorubicin, CA: carnosic acid, Vit E: vitamin E, LVDP: left ventricular developed pressure

**Table 4:** Doxorubicin and carnosic acid effects on body weight, heart weight, and the ratio of heart weight/body weight in control, doxorubicin, carnosic acid, and vitamin E treated groups

Groups	Bodyweight (g)	Heart weight (g)	Heart weight/body weight ratio (×10^-3^)
Control	269.25±13.1	0.90±0.02	3.1±0.06
CA (40 mg/kg)	288.75±8.25	0.91±0.01	3.1±0.09
DOX (2 mg/kg)	291.50±10.50	1.11±.06 #	3.9 ±0.23 #
DOX + CA (10 mg/kg)	282.75+19.98	0.88±0.02 *	3.1±0.14 *
DOX + CA (20 mg/kg)	284.00±13.34	0.86±0.05 *	3.0±0.10 *
DOX + CA (40 mg/kg)	294.75±16.02	0.84±0.03 *	2.8±0.08 *
DOX + Vit E (200 mg/kg)	320.00±8.01	0.95±0.03	2.9 ±0.10 *

The data were analyzed using ANOVA followed by Tukey’s *post-hoc* test. Each value represents the mean±SEM, with n=6 animals per group

^#^ compared to the control group; ^*^ compared to the DOX group

DOX: doxorubicin, CA: carnosic acid, Vit E: vitamin E

Tissue segments were stained with trichrome that shows muscle tissue in red, and collagen in blue (Trichrome×200). A) NS, B) CA (40 mg/kg, C) DOX-induced cardiomyopathy is characterized by an extensive amount of collagen deposition, D) DOX-induced cardiomyopathy is revealed by degeneration of myocardial fibers and cytoplasmic vacuolization, E) DOX+CA (10 mg/kg), F) DOX+CA (20 mg/kg), G) DOX+CA (40 mg/kg), H) DOX+Vit E (200 mg/kg)

Control group (NS), carnosic acid (CA), DOX (doxorubicin), Vitamin E (Vit E)

Data are expressed as median with interquartile range (IQR) (n=6). Statistical analysis was performed by non-parametric Kruskal-Wallis test and Dunn’s post-test. ^#^ compared with the control group, ^*^ compared with the DOX group

Control group (NS), carnosic acid (CA), DOX (doxorubicin), Vitamin E (Vit E)

DOX (2 mg/kg), carnosic acid (10, 20, and 40 mg/kg), and vitamin E (200 mg/kg) were injected IP into animals. The data is Mean±SD (n=6). ANOVA and post-test Tukey-Kramer were used for statistical analysis. ^#^ compared with the control group, ^*^ compared with the DOX group

Control group (NS), carnosic acid (CA), DOX (doxorubicin), Vitamin E (Vit E)

A) Specific bands for the Bax/Bcl-2 ratio have been investigated using western Blot. B) Bax/Bcl 2 ratio data by densitometric analysis. C) specific bands for pro and cleaved caspase-3 levels have been investigated using western Blot. D) Pro and cleaved caspase-3 level data by densitometric analysis. The data is Mean±SD (n=5). ANOVA and Post-test Tukey-Kramer were used for statistical analysis. ^#^ compared with the control group, ^*^ compared with the DOX group

Control group (NS), carnosic acid (CA), DOX (doxorubicin), Vitamin E (Vit E)

A) Specific bands for pro and cleaved caspase-8 levels have been investigated using western Blot. B) pro and cleaved caspase-8 level data by densitometric analysis. C) specific bands for the cleaved caspase-9 level have been investigated using western Blot. D) Cleaved caspase-9 level data by densitometric analysis. The data is Mean±SD (n=5). ANOVA and Post-test Tukey-Kramer were used for statistical analysis. ^#^ compared with the control group, ^*^ compared with the DOX group

Control group (NS), carnosic acid (CA), DOX (doxorubicin), Vitamin E (Vit E)

A) specific bands for IL-1β level have been investigated using western Blot. B) IL-1β level data by densitometric analysis. C) specific bands for TNF-α levels have been investigated using Western Blot. D) TNF-α level data by densitometric analysis. The data is Mean±SD (n=5). ANOVA and post-test Tukey-Kramer were used for statistical analysis. ^#^ compared with the control group, ^*^ compared with the DOX group.

Control group (NS), carnosic acid (CA), DOX (doxorubicin), Vitamin E (Vit E)

# compared to the control group

of MCF cells after 24 hr exposure

The data is Mean±SD (n=4). ANOVA and post-test Tukey-Kramer were used for statistical analysis. ^# ^compared with the control group

Carnosic acid (CA)

of MCF7 cells exposed to DOX (2 μM) for 24 hr

The data is Mean±SD (n=4). ANOVA and post-test Tukey-Kramer were used for statistical analysis. # compared with the control group, * compared to the DOX group

Carnosic acid (CA), DOX (doxorubicin)

## Conclusion

The obtained data reveals that DOX injection results in ECG abnormalities, disturbs hemodynamic parameters, and induces pathological alterations in rats’ cardiac tissue. Furthermore, DOX injection is followed by an enhancement in oxidative stress, inflammation, as well apoptosis in the heart. However, the administration of carnosic acid along with DOX prevents the alterations induced by DOX in ECG and hemodynamic parameters besides cardiac tissue through its anti-apoptotic, anti-inflammatory, and anti-oxidant properties. On the other hand, the findings of the* in vitro* part of the study unveiled that carnosic acid (5 and 10 μM) does not affect DOX properties as a chemotherapy medicine. Therefore, carnosic acid administration might be applied to treat or prevent DOX-induced cardiotoxicity in the future. However, more clinical investigations are required to confirm its efficiency.

## Authors’ Contributions

H H and BM R supervised the study; M GR administered the drugs, performed ECG, helped in hemodynamic surgery, isolation of the hearts, oxidative stress tests, analyzing the obtained data, and writing the manuscript; F E performed hemodynamic surgery (catheter Millar) and *in vitro* test and its analysis; A TY performed pathological evaluation; and M R wrote the proposal. All authors read and approved the final manuscript. The authors declare that all data were generated in-house and that no paper mill was used.

## Funding

This research was supported by the Pharmaceutical Research Center and the Vice-Chancellor of Research, Mashhad University of Medical Sciences, Iran.

## Ethics declaration

The authors complied with all ethical standards of scientific conduct.

## Conflicts of Interest

The authors declare that they have no conflicts of interest.
